# Combined predictive value of triglyceride-glucose index and remnant cholesterol for coronary artery disease in young adults

**DOI:** 10.3389/fcvm.2025.1651935

**Published:** 2025-10-29

**Authors:** Xi Wu, Mingxing Wu, Haobo Huang, Zhe Liu, He Huang, Lei Wang

**Affiliations:** Department of Cardiology, Xiangtan Central Hospital (The Affiliated Hospital of Hunan University), Xiangtan, Hunan, China

**Keywords:** coronary artery disease, young adults, triglyceride-glucose index, remnant cholesterol, cardiovascular risk stratification, atherosclerosis biomarkers

## Abstract

**Introduction:**

Coronary artery disease (CAD) is increasingly prevalent in individuals aged ≤45 years, yet effective early detection strategies remain lacking. Insulin resistance (IR) and remnant cholesterol (RC) burden are critical contributors to early atherosclerosis, highlighting the potential utility of novel markers such as the triglyceride-glucose (TyG) index and RC.

**Methods:**

In this retrospective study, we analyzed 458 patients aged ≤45 years who underwent coronary angiography (CAG). The TyG index and RC were calculated using standard fasting plasma glucose (FPG) and lipid profiles. Participants were classified into normal coronary, CAD, single-vessel disease (SVD), and multi-vessel disease (MVD) groups. Logistic regression analysis was performed to identify independent predictors of CAD and MVD. Receiver operating characteristic (ROC) curves were used to evaluate the discriminative ability of TyG and RC.

**Results:**

Both the TyG index and RC were significantly higher in patients with CAD compared with controls (*P* < 0.001), and further elevated in those with MVD. In multivariate analysis, TyG (odds ratio [OR] = 1.393) and RC (OR = 1.475) were independently associated with CAD and also predicted MVD (TyG OR = 2.363; RC OR = 3.692). RC had a higher area under the curve (AUC) for identifying CAD than TyG (0.773 vs. 0.669), whereas TyG had greater predictive value for MVD (AUC = 0.775 vs. 0.683).

**Discussion:**

The TyG index and RC are independent, complementary, and accessible biomarkers for assessing early CAD risk in young adults. RC showed higher sensitivity in identifying the presence of CAD, while higher TyG index values were associated with multi-vessel involvement. Incorporating these markers into screening protocols may enhance early risk stratification and help prevent premature atherosclerosis in young populations.

## Introduction

Premature coronary artery disease (CAD) in young adults is a rising but under-recognized public health issue. Most risk stratification strategies focus on older populations, leaving younger patients overlooked despite their higher lifetime disease burden. Identifying simple, non-invasive biomarkers for early detection in this group remains an urgent unmet need. CAD, characterized by the progression of atherosclerotic plaques, lipid accumulation, and vascular calcification, continues to be a primary contributor to global morbidity and mortality. In the United States, approximately 20.5 million individuals are affected, with prevalence rates reaching 10.9% among adults aged ≥45 years and 17.0% in those ≥65 years (1). Notably, the age of disease onset has been shifting downward, rendering premature CAD an escalating public health issue. Young patients diagnosed with CAD are at risk of enduring a prolonged disease trajectory due to early atherosclerosis and limited access to preventive care strategies (2). These evolving patterns underscore the urgent need to identify effective, non-invasive biomarkers that facilitate early risk stratification in this emerging population.

Although conventional risk factors such as smoking, dyslipidemia, obesity, hypertension, and hyperglycemia remain critical, increasing attention has been directed toward insulin resistance (IR) as a central metabolic disturbance in CAD development. IR contributes to pathophysiological changes by disturbing lipid and glucose regulation, impairing vascular endothelial and smooth muscle cell functions, and promoting chronic systemic inflammation (3). These mechanisms suggest that IR may be involved in the early development of atherosclerotic lesions among younger individuals.

The triglyceride-glucose (TyG) index, derived from fasting levels of triglycerides (TG) and glucose, has been validated as a practical and efficient proxy for assessing IR. Numerous studies have indicated that the TyG index surpasses the homeostasis model assessment of insulin resistance (HOMA-IR) in predicting IR at the population level (4). Additionally, TyG has shown consistent associations with CAD onset, progression, and adverse outcomes (5, 6). Emerging data also suggest that TyG independently correlates with the burden of coronary lesions as quantified by angiographic scoring systems (7, 8). However, the existing literature predominantly focuses on older cohorts, leaving a significant knowledge gap regarding TyG's utility in younger patients, despite the rising incidence of CAD in this demographic.

Remnant cholesterol (RC) refers to the cholesterol content of triglyceride-rich lipoproteins (TRLs). These particles include very-low-density lipoprotein (VLDL), intermediate-density lipoprotein (IDL), and chylomicron remnants. RC has recently gained attention as a potent atherogenic factor (9). Unlike TG, RC reflects the cholesterol-rich portion of TRLs and demonstrates a stronger link to cardiovascular events (10). RC-carrying particles are larger, more cholesterol-dense, and exhibit heightened pro-inflammatory properties compared to low-density lipoprotein cholesterol (LDL-C). Their pathogenic potential is mediated through endothelial dysfunction, oxidative stress, and foam cell formation (11, 12). Moreover, RC is closely related to conditions such as diabetes and hypertension and may account for residual cardiovascular risk even in patients with optimally controlled LDL-C levels (13). The observed discordance between RC and LDL-C in metabolic syndrome further highlights the limitations of LDL-C-centered risk models (14).

Taken together, TyG and RC offer insights into distinct but complementary domains of cardiometabolic risk: the former reflecting IR-associated glucose-lipid dysregulation, and the latter indicating cholesterol-mediated atherogenesis. However, few studies have assessed their combined value in evaluating coronary lesion severity or predicting multi-vessel involvement, particularly in young adults—a population increasingly affected by early-onset CAD yet often excluded from cardiovascular research. Addressing this gap, the present study aimed to investigate the individual and combined associations of the TyG index and RC with the presence and angiographic severity of CAD in young adults, and to evaluate their potential as complementary, non-invasive biomarkers for early identification and stratification in this high-risk group.

## Materials and methods

### Study participants

**Figure 1:**
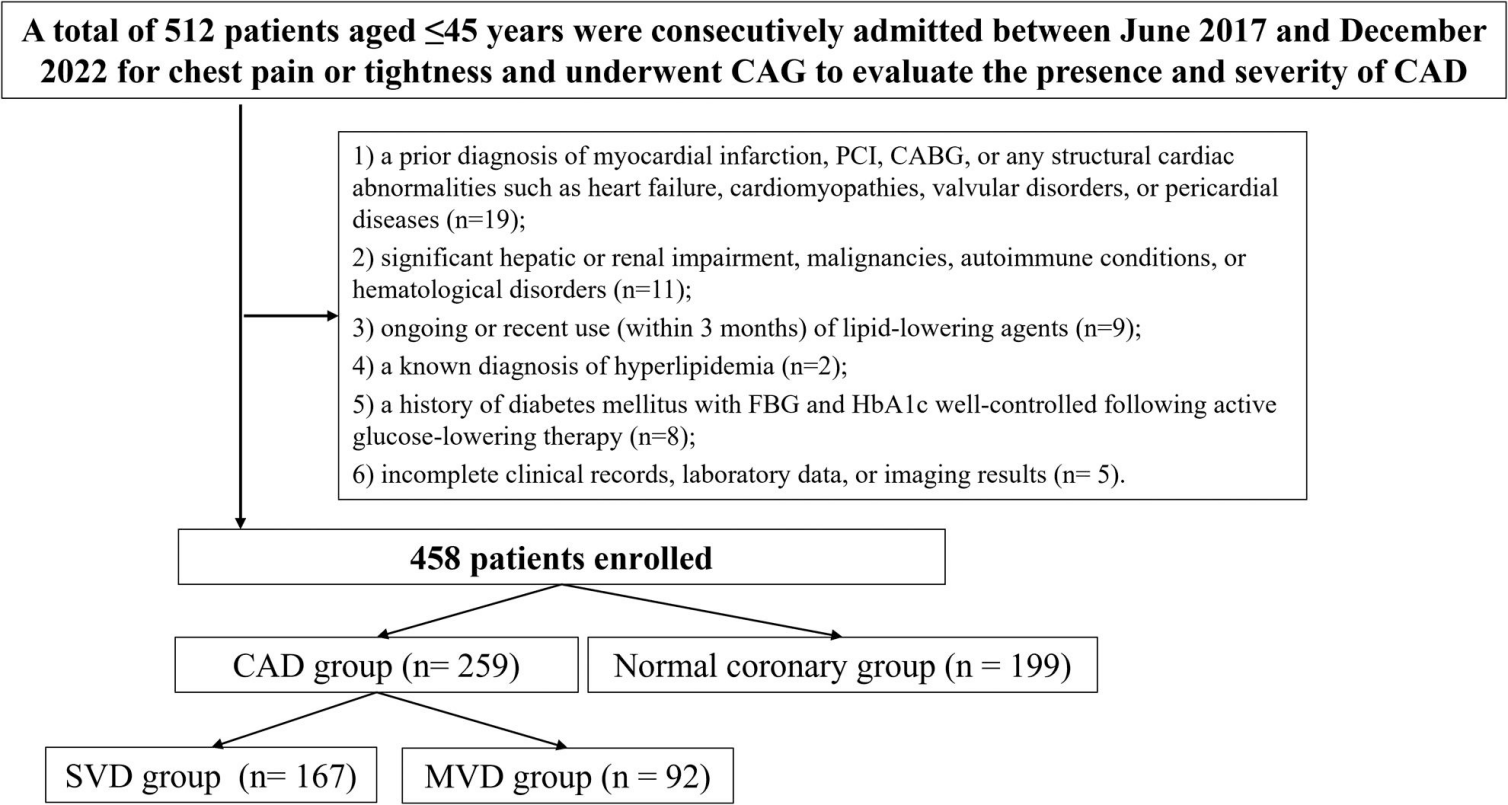
Study flowchart. SVD, single-vessel disease; MVD, multi-vessel disease; CAD, Coronary artery disease; PCI, percutaneous coronary intervention; CABG, coronary artery bypass grafting; FBG, fasting blood glucose; HbA1c, hemoglobin A1c; CAG, coronary angiography.

This retrospective, single-center observational study was carried out at the Department of Cardiology, Xiangtan Central Hospital. Patients aged 45 years or younger who were consecutively admitted between June 2017 and December 2022 for evaluation of chest pain or tightness and underwent CAG to assess the presence and severity of CAD were considered for inclusion. The eligibility criteria were as follows (1): age ≤45 years; (2) clinical symptoms including chest pain or tightness indicative of possible CAD; and (3) availability of comprehensive clinical data encompassing demographics, laboratory results, and CAG findings. Exclusion criteria included: (1) a prior diagnosis of myocardial infarction, percutaneous coronary intervention (PCI), coronary artery bypass grafting (CABG), or any structural cardiac abnormalities such as heart failure, cardiomyopathies, valvular disorders, or pericardial diseases; (2) significant hepatic or renal impairment, malignancies, autoimmune conditions, or hematological disorders; (3) ongoing or recent use (within 3 months) of lipid-lowering agents; (4) a known diagnosis of hyperlipidemia; (5) a history of diabetes mellitus with fasting blood glucose (FBG) and hemoglobin A1c (HbA1c) well-controlled following active glucose-lowering therapy; and (6) incomplete clinical records, laboratory data, or imaging results (Figure 1). The study protocol received approval from the Ethics Committee of Xiangtan Central Hospital (Approval No. X2018762) and was conducted in alignment with the ethical standards outlined in the Declaration of Helsinki (2013 revision). Written informed consent was obtained from all participants. In instances where written consent was not practicable, verbal consent was documented in the patients’ electronic medical records by the attending physician, in accordance with institutional ethical guidelines.

### Data collection

Demographic and clinical characteristics were extracted from the hospital's electronic medical record system. Variables collected included age, sex, smoking and alcohol use history, prior diagnoses of hypertension and diabetes mellitus, body mass index (BMI), systolic blood pressure (SBP), heart rate, left ventricular ejection fraction (LVEF), and left ventricular end-diastolic diameter (LVEDD). Laboratory data were obtained from fasting venous blood samples collected on the morning of the second day of hospitalization, following a minimum 12 h overnight fast. All specimens were processed by qualified personnel in the hospital's clinical laboratory. Standard hematological and biochemical assessments comprised white blood cell (WBC) count, red blood cell (RBC) count, platelet count (PLT), fasting plasma glucose (FPG), total cholesterol (TC), TG, low-density lipoprotein cholesterol (LDL-C), high-density lipoprotein cholesterol (HDL-C), alanine aminotransferase (ALT), aspartate aminotransferase (AST), serum creatinine (SCr), and blood urea nitrogen (BUN).

The TyG index was calculated using the following formula (15):

TyGindex=ln[TG(mg/dL)×FPG(mg/dL)/2]

The RC was estimated indirectly using the following equation (16):

RC=TC−HDL-C−LDL-C

### Coronary angiography and lesion assessment

All patients underwent CAG during their hospital stay, utilizing the conventional Judkins technique. CAD was defined as a luminal diameter reduction of ≥50% in any of the three principal coronary arteries—namely, the left anterior descending (LAD), left circumflex (LCX), or right coronary artery (RCA)—or a stenosis of ≥40% in the left main (LM) artery, as determined by angiographic evaluation. Individuals not meeting these diagnostic thresholds were classified into the control group. Based on angiographic results, single-vessel disease (SVD) was identified when only one of the three major epicardial vessels exhibited ≥50% stenosis. Multi-vessel disease (MVD) was characterized by ≥50% narrowing in two or more primary coronary arteries, or the involvement of LM disease irrespective of additional vessel stenosis. CAG was performed using a GE Innova 3100 digital angiography system (GE Healthcare, Chicago, IL, USA). Quantitative coronary angiography (QCA) was conducted with the QAngio XA software (version XX, Medis Medical Imaging Systems, Leiden, the Netherlands).

### Study grouping

A total of 458 eligible participants were included in the final analysis, comprising 259 patients with confirmed CAD group and 199 individuals with normal coronary angiographic findings (normal coronary group). Among those in the CAD group, patients were further stratified based on the number of affected vessels and the severity of coronary lesions. According to the number of diseased vessels, 167 patients were classified into the SVD group and 92 into the MVD group.

### Statistical analysis

All statistical analyses were performed using SPSS version 25.0 (IBM Corp., Armonk, NY, USA). Continuous variables with a normal distribution were expressed as mean ± standard deviation (SD), and comparisons between groups were conducted using independent-samples t-tests. For non-normally distributed continuous data, results were presented as medians with interquartile ranges (IQRs), and differences were assessed using the Mann–Whitney *U* test (rank-sum test). Categorical variables were summarized as frequencies and percentages, and between-group comparisons were evaluated using the chi-square (*χ*^2^) test. Correlation analyses were conducted using Pearson's correlation coefficient when both variables followed a normal distribution; otherwise, Spearman's rank correlation was applied if one or both variables were non-normally distributed. To construct the multivariate logistic regression models, variables with *P* < 0.05 in univariate analyses were selected as candidate predictors. Variables that are mathematically or biologically derived from one another—such as triglycerides and fasting glucose for the TyG index, or total cholesterol and LDL-C for RC—were not included simultaneously in the same model to reduce potential collinearity. Although the variance inflation factor (VIF) was not formally calculated, careful attention was paid to variable selection to avoid redundancy and ensure model stability. Binary logistic regression analysis was performed to identify independent risk factors associated with CAD and multi-vessel coronary artery disease. Receiver operating characteristic (ROC) curves were constructed to evaluate the predictive performance and determine the optimal cutoff values of the TyG index and RC. ROC curves were constructed to evaluate the predictive performance and determine the optimal cutoff values of the TyG index and RC. ROC curves plot sensitivity (true positive rate) against 1-specificity (false positive rate) at various threshold settings. The area under the curve (AUC) provides a quantitative measure of overall diagnostic accuracy, ranging from 0.5 (no discrimination) to 1.0 (perfect discrimination). Sensitivity refers to the proportion of actual positives correctly identified, while specificity reflects the proportion of actual negatives correctly classified. The optimal cutoff point was determined by maximizing the Youden index (sensitivity + specificity-1), which balances both sensitivity and specificity. A two-tailed *P* value of <0.05 was considered statistically significant. This two-tiered grouping strategy was designed to first compare the presence of CAD (control vs. CAD) and subsequently assess disease severity (SVD vs. MVD) within the CAD cohort. Direct three-group comparisons were not performed in order to maintain statistical clarity and avoid over-fragmentation of the dataset.

## Results

### Baseline characteristics of the study population

A total of 458 young adults (age ≤45 years) were enrolled in the study, including 259 patients diagnosed with CAD group and 199 individuals with angiographically normal coronary arteries (normal coronary group). All angiographic images were independently interpreted by two experienced interventional cardiologists (X.W. and L.W.), both blinded to patients’ clinical and laboratory information. Agreement between observers and within the same observer for lesion categorization was excellent, with Cohen's κ coefficients of 0.90 and 0.93, respectively. The baseline demographic and clinical characteristics of the two groups are summarized in Table 1.

**Table 1:** Baseline clinical characteristics and laboratory biomarkers.

Variables	ALL(*n* = 458)	CAD group (*n* = 259)	Normal coronary group (*n* = 199)	*P* value
Age (years)	40 (38, 42)	40 (37, 42)	40 (38, 42)	0.716
Male, *n*%	273 (59.6%)	157 (60.6%)	116 (58.3%)	0.684
Hypertension, *n*%	215 (46.9%)	116 (44.8%)	99 (49.7%)	0.337
Smoking history, *n*%	134 (29.3%)	78 (30.1%)	56 (28.1%)	0.721
Previous stroke, *n*%	22 (4.8%)	17 (6.6%)	5 (2.5%)	0.073
Previous heart failure, *n*%	13 (2.8%)	6 (2.3%)	7 (3.5%)	0.628
Peripheral vascular disease, n%	15 (3.3%)	8 (3.1%)	7 (3.5%)	1.000
Body mass index, kg/m^2^	25.11 ± 2.72	25.23 ± 2.51	24.92 ± 2.84	0.322
SBP, mmHg	128.31 ± 24.73	130.01 ± 23.34	126.11 ± 26.25	0.094
Heart rate, bpm	76.42 ± 14.94	77.03 ± 14.85	75.74 ± 15.07	0.367
LVEF, %	52.83 ± 6.52	52.85 ± 6.33	52.81 ± 6.76	0.990
LVEDD, mm	49.51 ± 5.76	49.44 ± 5.46	49.74 ± 6.07	0.491
Laboratory biomarkers				
AST, U/L	23.14 (20.65, 25.56)	23.36 (20.75, 25.83)	22.53 (20.48, 25.15)	0.128
ALT, U/L	48.09 (42.68, 53.68)	48.28 (43.52, 53.76)	47.78 (41.82, 53.35)	0.362
Scr, umo/L	88.96 (85.47, 92.44)	88.97 (85.58, 92.39)	88.87 (85.39, 92.53)	0.838
BUN, mmol/L	4.99 (4.57, 5.43)	4.98 (4.55, 5.46)	4.99 (4.59, 5.41)	0.864
WBC, 10^9 ^/L	7.77 (6.30, 9.80)	9.38 (8.01, 10.78)	6.34 (5.49, 7.16)	<0.001
RBC, 10^12 ^/L	4.96 (4.85, 5.05)	4.96 (4.85, 5.05)	4.97 (4.85, 5.05)	0.997
PLT, 10^9 ^/L	246.84 (237.49, 258.97)	245.85 (236.91, 257.63)	249.32 (238.57, 260.13)	0.174
FPG, mmol/L	5.47 ± 0.48	5.60 ± 0.48	5.29 ± 0.41	<0.001
HbA1C (%)	5.86 ± 0.61	5.86 ± 0.58	5.86 ± 0.65	0.981
TG, mmol/L	1.70 (1.33, 1.99)	1.84 (1.51, 2.12)	1.57 (1.26, 1.80)	<0.001
TC, mmol/L	4.38 (3.89, 4.87)	4.52 (4.04, 5.01)	4.20 (3.74, 4.64)	<0.001
HDL, mmol/L	1.12 (0.98, 1.28)	1.07 (0.93, 1.23)	1.19 (1.04, 1.34)	<0.001
LDL, mmol/L	2.58 (2.23, 2.96)	2.69 (2.29, 3.10)	2.50 (2.15, 2.81)	<0.001
Lp(a), mmol/L	197.87 (178.79, 219.61)	196.44 (180.26, 219.59)	198.70 (176.56, 219.59)	0.649
ApoA1, g/L	1.19 ± 0.35	1.18 ± 0.38	1.19 ± 0.31	0.751
ApoB, g/L	0.93 ± 0.24	0.91 ± 0.23	0.95 ± 0.24	0.090
TBIL, umol/L	16.41 (15.21, 17.63)	16.37 (15.05, 17.60)	16.53 (15.45, 17.73)	0.491
Uric acid, umol/L	474.29 (444.56, 502.21)	474.60 (444.15, 501.34)	473.13 (445.70, 502.91)	0.922
TyG index	8.83 (8.47, 9.17)	8.96 (8.60, 9.37)	8.65 (8.41, 8.95)	<0.001
RC, mmol\L	0.70 (0.52, 0.88)	0.80 (0.65, 0.97)	0.57 (0.43, 0.71)	<0.001

Continuous variables were expressed as mean ± SD, or median (interquartile range). Categorical variables were expressed as number (percentage).

SBP, systolic blood pressure; LVEF, left ventricular ejection fraction; LVEDD, left ventricular end-diastolic diameter; CAD, coronary artery disease; LVEF, left ventricular ejection fraction; LVEDD, left ventricular end-diastolic diameter; TG, triglycerides; TC, total cholesterol; HDL, high-density lipoprotein; LDL, low-density lipoprotein; Lp(a), lipoprotein(a); AST, aspartate aminotransferase; ALT, alanine aminotransferase; TBIL, total bilirubin; Scr, serum creatinine; BUN, blood urea nitrogen; WBC, white blood cell; RBC, red blood cell; PLT, platelet count; FPG, fasting plasma glucose; HbA1C, hemoglobin A1c; ApoA1, apolipoprotein A1; ApoB, apolipoprotein B; TyG, triglyceride-glucose index; RC, remnant cholesterol; Lp(a), lipoprotein(a).

RC was measured in mmol/L. To convert to mg/dl, multiply by 38.67.

**Figure 2:**
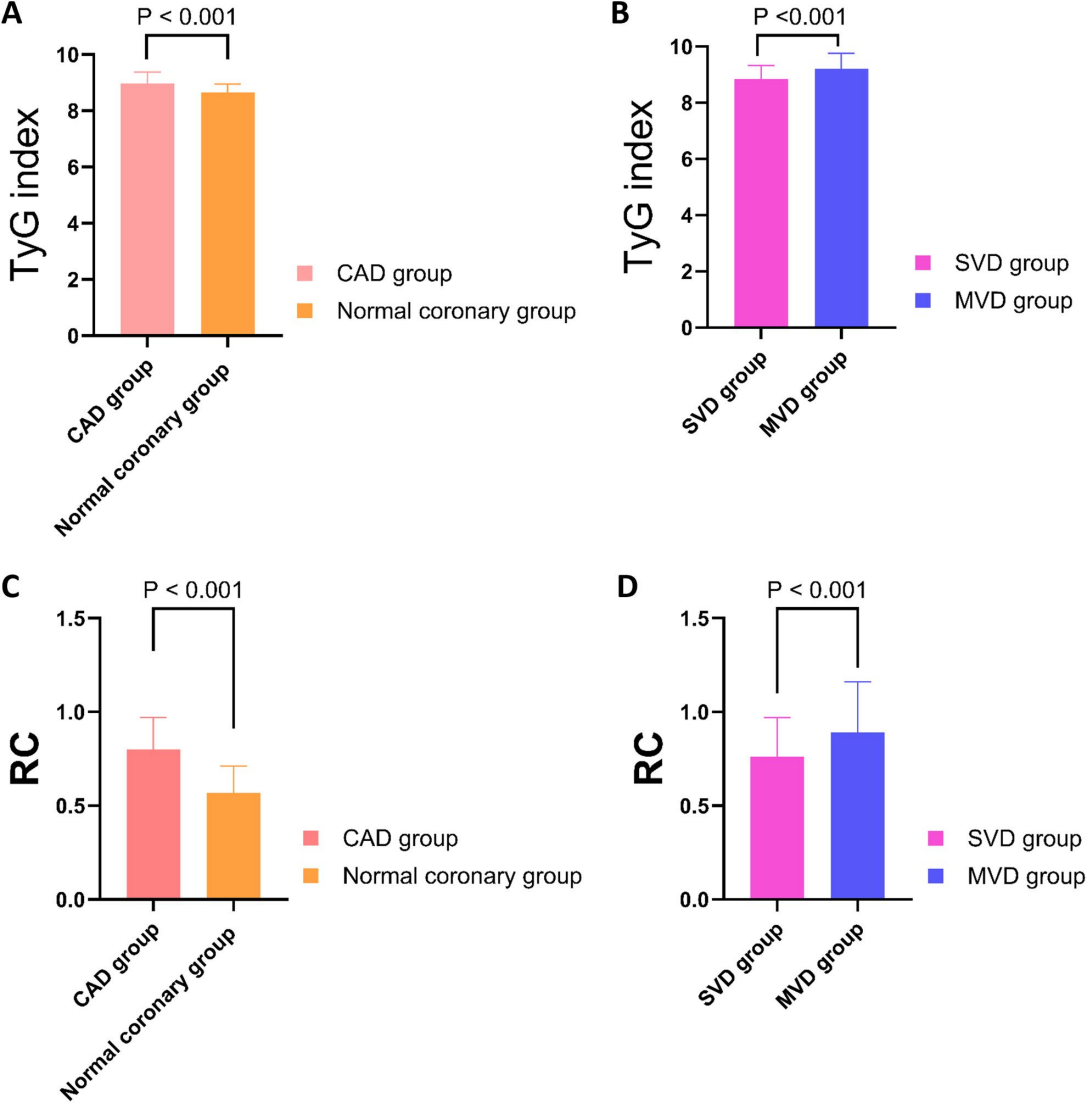
Comparison of TyG index and RC levels among different groups. **(A)** The TyG index was significantly higher in the CAD group compared to the normal coronary group (*P* < 0.001). **(B)** Within the CAD cohort, the TyG index was significantly elevated in the MVD group compared to the SVD group (*P* < 0.001). **(C)** RC levels were significantly higher in the CAD group than in the normal coronary group (*P* < 0.001). **(D)** RC levels were significantly increased in the MVD group relative to the SVD group (*P* < 0.001). TyG, triglyceride-glucose index; RC, remnant cholesterol; CAD, Coronary artery disease; SVD, single-vessel disease; MVD, multi-vessel disease.

There were no significant differences in age, sex distribution, BMI, blood pressure, or heart function parameters (LVEF, LVEDD) between the two groups (all *P* > 0.05). Traditional cardiovascular risk factors such as hypertension, smoking history, previous stroke, and peripheral vascular disease were also similar between groups (*P* > 0.05). However, key metabolic indicators exhibited statistically significant differences. The CAD group had higher levels of fasting plasma glucose (FPG: 5.60 ± 0.48 mmol/L vs. 5.29 ± 0.41 mmol/L, *P* < 0.001) and TG [1.84 [1.51–2.12] vs. 1.57 [1.26–1.80] mmol/L, *P* < 0.001] than the normal coronary group. This was accompanied by lower levels of HDL-C [1.07 [0.93–1.23] vs. 1.19 [1.04–1.34] mmol/L, *P* < 0.001]. Importantly, the TyG index was significantly elevated in the CAD group compared with controls [8.96 [8.60–9.37] vs. 8.65 [8.41–8.95], *P* < 0.001], indicating a higher degree of insulin resistance (Figure 2A). Similarly, RC levels were also higher in the CAD group [0.80 [0.65–0.97] vs. 0.57 [0.43–0.71] mmol/L, *P* < 0.001], suggesting increased atherogenic lipid burden(Figure 2C). In addition, the CAD group exhibited markedly elevated white blood cell counts [WBC: 9.38 [8.01–10.78] vs. 6.34 [5.49–7.16] × 10⁹ /L, *P* < 0.001], indicating heightened systemic inflammation.

### Logistic regression analysis of predictors for coronary artery disease

In the multivariate logistic regression model (Table 2), which adjusted for potential confounders, TyG index remained a statistically significant independent predictor [odds ratios [OR]: 1.393, 95% confidence interval [CI]: 1.143–1.763, *P* = 0.019], as did RC (OR: 1.475, 95% CI: 1.264–1.813, *P* = 0.012). Interestingly, LDL-C lost its statistical significance in the multivariate model (OR: 1.172, 95% CI: 0.944–1.466, *P* = 0.074), suggesting that RC and TyG may better capture residual cardiometabolic risk in this younger population.

**Table 2:** Univariate and multivariate Cox regression analyses showing independent predictors of CAD.

Variables	Univariate analysis			Multivariate analysis		
	OR	95% CI	*P* value	OR	95% CI	*P* value
WBC, 10^9 ^/L	1.085	0.874–1.344	0.247			
FPG, mmol/L	1.144	0.964–1.423	0.186			
TG, mmol/L	1.096	0.885–1.364	0.229			
TC, mmol/L	1.133	0.911–1.397	0.194			
HDL, mmol/L	1.064	0.854–1.375	0.267			
LDL, mmol/L	1.712	1.453–2.062	0.021	1.172	0.944–1.466	0.074
TyG index	1.434	1.122–1.835	0.017	1.393	1.143–1.763	0.019
RC, mmol/L	1.523	1.223–1.895	0.009	1.475	1.264–1.813	0.012

CAD, coronary artery disease; OR, odds ratios; CI, confidence interval; WBC, white blood cell; FPG, fasting plasma glucose; TG, triglycerides; TC, total cholesterol; HDL, high-density lipoprotein; LDL, low-density lipoprotein; TyG, triglyceride-glucose index; RC, remnant cholesterol.

RC was measured in mmol/L. To convert to mg/dl, multiply by 38.67.

### Comparison between single-vessel and multi-vessel disease groups

Among the 259 young patients diagnosed with CAD, 167 were classified into the SVD group and 92 into the MVD group (Table 3). Basic demographic parameters including age, gender distribution, SBP, heart rate, and cardiac function (LVEF and LVEDD) showed no statistically significant differences between the two groups (*P* > 0.05). Similarly, the prevalence of hypertension, smoking history, and previous stroke did not differ significantly. However, several important laboratory biomarkers demonstrated marked elevations in the MVD group, reflecting increased cardiometabolic burden. FPG was significantly higher in patients with MVD compared to those with SVD (5.73 ± 0.44 mmol/L vs. 5.44 ± 0.48 mmol/L, *P* < 0.001), as was HbA1C (5.95% ± 0.60% vs. 5.75 ± 0.54%, *P* = 0.005), indicating poorer glycemic status. Likewise, serum TG (2.15 ± 0.33 mmol/L vs. 1.40 ± 0.35 mmol/L, *P* < 0.001) and TC [5.24 [4.97, 5.54] vs. 4.17 [3.79, 4.47] mmol/L, *P* < 0.001] were substantially higher in the MVD group, as were LDL-C and apolipoprotein B (ApoB), the latter being a marker of atherogenic particle burden (*P* = 0.041 and *P* = 0.018, respectively). Of note, both the TyG index and RC levels were significantly elevated in the MVD group. Specifically, TyG index values averaged 9.21 ± 0.55 in MVD patients vs. 8.84 ± 0.49 in those with SVD (*P* < 0.001) (Figure 2B), and RC concentrations were 0.89 ± 0.27 mmol/L vs. 0.76 ± 0.21 mmol/L (*P* < 0.001) (Figure 2D). These findings reinforce the association of these two markers with more severe coronary involvement. In addition, Scr and total bilirubin (TBIL) were also significantly higher in the MVD group, possibly reflecting greater systemic stress or comorbid conditions (*P* < 0.001 for both). No significant differences were observed in HDL-C, uric acid, apolipoprotein A1(ApoA1), or white blood cell counts.

**Table 3:** Comparison of baseline characteristics and laboratory biomarkers between the single-vessel and multi-vessel disease groups.

Variables	ALL (*n* = 259)	SVD group (*n* = 167)	MVD group (*n* = 92)	*P* value
Age (years)	40 (37, 42)	39 (37, 41)	39 (37, 41)	0.726
Male, *n* %	157 (60.6%)	97 (58.1%)	60 (65.2%)	0.321
Hypertension, *n* %	116 (44.8%)	78 (46.7%)	38 (41.3%)	<0.001
Smoking history, *n* %	78 (30.1%)	51 (30.5%)	27 (29.3%)	<0.001
Previous stroke, *n* %	17 (6.6%)	9 (5.4%)	8 (8.7%)	0.443
Previous heart failure, *n* %	6 (2.3%)	6 (3.6%)	0 (0.0%)	0.159
Peripheral vascular disease, *n* %	8 (3.1%)	4 (2.4%)	4 (4.3%)	0.621
Body mass index, kg/m^2^	25.22 ± 2.58	25.22 ± 2.55	25.21 ± 2.60	0.968
SBP, mmHg	130.02 ± 23.35	129.38 ± 23.34	131.46 ± 23.43	0.489
Heart rate, bpm	77.03 ± 14.85	76.16 ± 15.44	78.63 ± 13.87	0.175
LVEF, %	52.82 ± 6.35	52.94 ± 6.56	52.71 ± 6.06	0.792
LVEDD, mm	49.44 ± 5.46	49.35 ± 5.26	49.61 ± 5.86	0.659
Laboratory biomarkers				
AST, U/L	23.36 ± 3.84	23.57 ± 3.88	23.08 ± 3.82	0.325
ALT, U/L	48.28 ± 8.10	48.27 ± 8.13	48.55 ± 7.79	0.781
Scr, umo/L	88.97 ± 2.63	85.91 ± 3.92	93.90 ± 2.33	<0.001
BUN, mmol/L	4.98 ± 0.62	4.96 ± 0.64	5.06 ± 0.63	0.242
WBC, 10^9 ^/L	9.38 ± 2.09	9.37 ± 2.15	9.47 ± 1.89	0.709
RBC, 10^12 ^/L	4.96 ± 0.14	4.95 ± 0.15	4.97 ± 0.14	0.379
PLT, 10^9 ^/L	247.19 ± 17.58	247.38 ± 16.56	247.03 ± 18.41	0.874
FPG, mmol/L	5.66 ± 0.48	5.44 ± 0.48	5.73 ± 0.44	<0.001
HbA1C (%)	5.86 ± 0.58	5.75 ± 0.54	5.95 ± 0.60	0.005
TG, mmol/L	1.82 ± 0.51	1.40 ± 0.35	2.15 ± 0.33	<0.001
TC, mmol/L	4.52 (4.04, 5.01)	4.17 (3.79, 4.47)	5.24 (4.97, 5.54)	<0.001
HDL, mmol/L	1.07 (0.93, 1.23)	1.07 (0.94, 1.23)	1.08 (0.91, 1.23)	0.907
LDL, mmol/L	2.69 (2.29, 3.10)	2.60 (2.21, 3.04)	2.81 (2.42, 3.16)	0.041
Lp(a), mmol/L	196.44 (180.26, 219.59)	196.00 (181.11, 219.26)	196.82 (178.51, 219.58)	0.730
ApoA1, g/L	1.18 ± 0.38	1.18 ± 0.37	1.18 ± 0.41	0.984
ApoB, g/L	0.91 ± 0.23	0.89 ± 0.22	0.96 ± 0.25	0.018
TBIL, umol/L	16.36 ± 1.74	15.35 ± 1.19	18.19 ± 0.89	<0.001
Uric acid, umol/L	473.70 ± 44.44	467.82 ± 45.01	485.10 ± 41.12	0.078
TyG index	8.97 ± 0.53	8.84 ± 0.49	9.21 ± 0.55	<0.001
RC, mmol/L	0.80 ± 0.24	0.76 ± 0.21	0.89 ± 0.27	<0.001

Continuous variables were expressed as mean ± SD, or median (interquartile range). Categorical variables were expressed as number (percentage).

SBP, systolic blood pressure; LVEF, left ventricular ejection fraction; LVEDD, left ventricular end-diastolic diameter; LVEF, left ventricular ejection fraction; LVEDD, left ventricular end-diastolic diameter; SVD, single-vessel disease; MVD, multi-vessel disease; TG, triglycerides; TC, total cholesterol; HDL, high-density lipoprotein; LDL, low-density lipoprotein; Lp(a), lipoprotein(a); AST, aspartate aminotransferase; ALT, alanine aminotransferase; TBIL, total bilirubin; Scr, serum creatinine; BUN, blood urea nitrogen; WBC, white blood cell; RBC, red blood cell; PLT, platelet count; FPG, fasting plasma glucose; HbA1C, hemoglobin A1c; ApoA1, apolipoprotein A1; ApoB, apolipoprotein B; TyG, triglyceride-glucose index; RC, remnant cholesterol; Lp(a), lipoprotein(a).

RC was measured in mmol/L. To convert to mg/dl, multiply by 38.67.

### Independent predictors of multi-vessel disease

In the multivariate regression model adjusting for potential confounders (Table 4), both TyG index and RC remained independent predictors of MVD. The TyG index demonstrated a strong association with the risk of multi-vessel disease (OR: 2.363, 95% CI: 1.379–3.582, *P* < 0.001), while RC showed an even stronger predictive power (OR: 3.692, 95% CI: 1.964–8.921, *P* = 0.001). Interestingly, LDL-C, although significant in univariate analysis, lost statistical significance in the multivariate model (OR: 2.531, 95% CI: 0.682–3.521, *P* = 0.640), indicating its inferior predictive value compared to RC and TyG.

**Table 4:** Univariate and multivariate Cox regression analyses showing independent predictors of MVD.

Variables	Univariate analysis			Multivariate analysis		
	OR	95% CI	*P* value	OR	95% CI	*P* value
Hypertension	1.323	0.645–3.170	0.453			
Smoking history	1.652	0.852–3.669	0.092			
Scr, umo/L	1.268	0.783–3.821	0.375			
FPG, mmol/L	1.226	0.721–2.875	0.842			
HbA1C (%)	1.643	0.771–2.849	0.593			
TG, mmol/L	1.675	0.774–4.582	0.181			
TC, mmol/L	1.421	0.781–1.763	0.664			
LDL, mmol/L	2.323	1.640–5.737	0.001	2.531	0.682–3.521	0.640
ApoB, g/L	1.023	0.445–2.335	0.602			
TBIL, umol/L	1.138	0.720–2.001	0.534			
TyG index	1.176	1.092–1. 381	<0.001	2.363	1.379–3.582	<0.001
RC, mmol/L	1.628	1.583–2.322	<0.001	3.692	1.964–8.921	0.001

MVD, multi-vessel disease; OR, odds ratios; CI, confidence interval; TyG, triglyceride-glucose index; RC, remnant cholesterol; TBIL, total bilirubin; ApoB, apolipoprotein B; LDL, low-density lipoprotein; TG, triglycerides; TC, total cholesterol; HbA1C, hemoglobin A1c; FPG, fasting plasma glucose; Scr, serum creatinine;.

RC was measured in mmol/L. To convert to mg/dl, multiply by 38.67.

### Diagnostic performance of TyG index and remnant cholesterol for CAD and MVD

**Figure 3:**
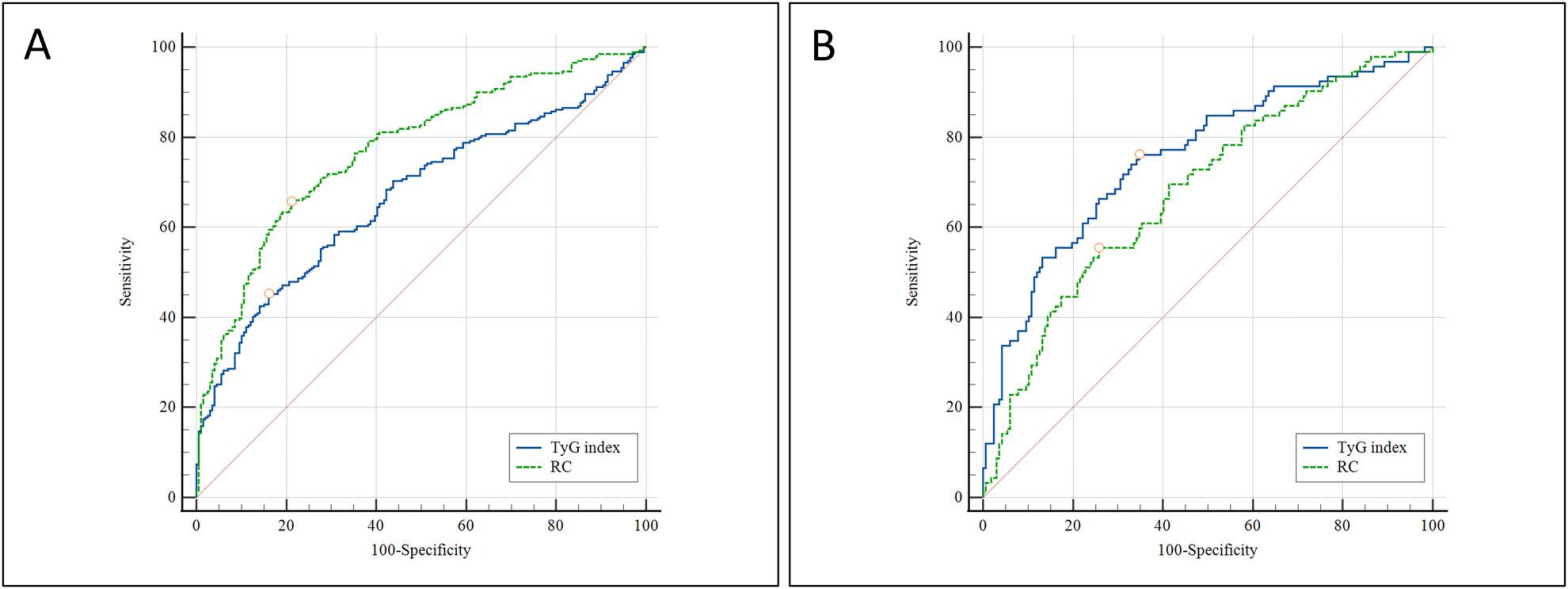
ROC curve analysis comparing the predictive value of the TyG index and RC for: **(A)** the presence of CAD; and **(B)** the presence of multi-vessel coronary artery disease. ROC, receiver operating characteristic; TyG, triglyceride-glucose index; RC, remnant cholesterol; CAD, Coronary artery disease;.

ROC curve analysis was conducted to evaluate the discriminative ability of the TyG index and RC in predicting the presence of CAD and MVD. For identifying the presence of CAD, the AUC for the TyG index was 0.669 (95% CI: 0.624–0.712, *P* < 0.001), indicating a statistically significant predictive value. The optimal cutoff value was 8.893, yielding a sensitivity of 45.2% and a specificity of 83.9%. In contrast, RC showed superior discriminative ability, with an AUC of 0.773 (95% CI: 0.732–0.811, *P* < 0.001). The optimal threshold for RC was 0.728 mmol/L, with a sensitivity of 65.6% and a specificity of 78.8% (Figure 3A). When predicting multi-vessel coronary artery disease, the TyG index again showed robust performance, with an AUC of 0.775 (95% CI: 0.698–0.807, *P* < 0.001). The optimal cutoff value was 9.137, corresponding to a sensitivity of 76.3% and a specificity of 65.2%. For RC, the AUC was 0.683 (95% CI: 0.622–0.739, *P* < 0.001), with an optimal cutoff of 0.877 mmol/L, yielding 55.6% sensitivity and 74.2% specificity (Figure 3B).

### Correlation between TyG index and RC in CAD patients

**Figure 4:**
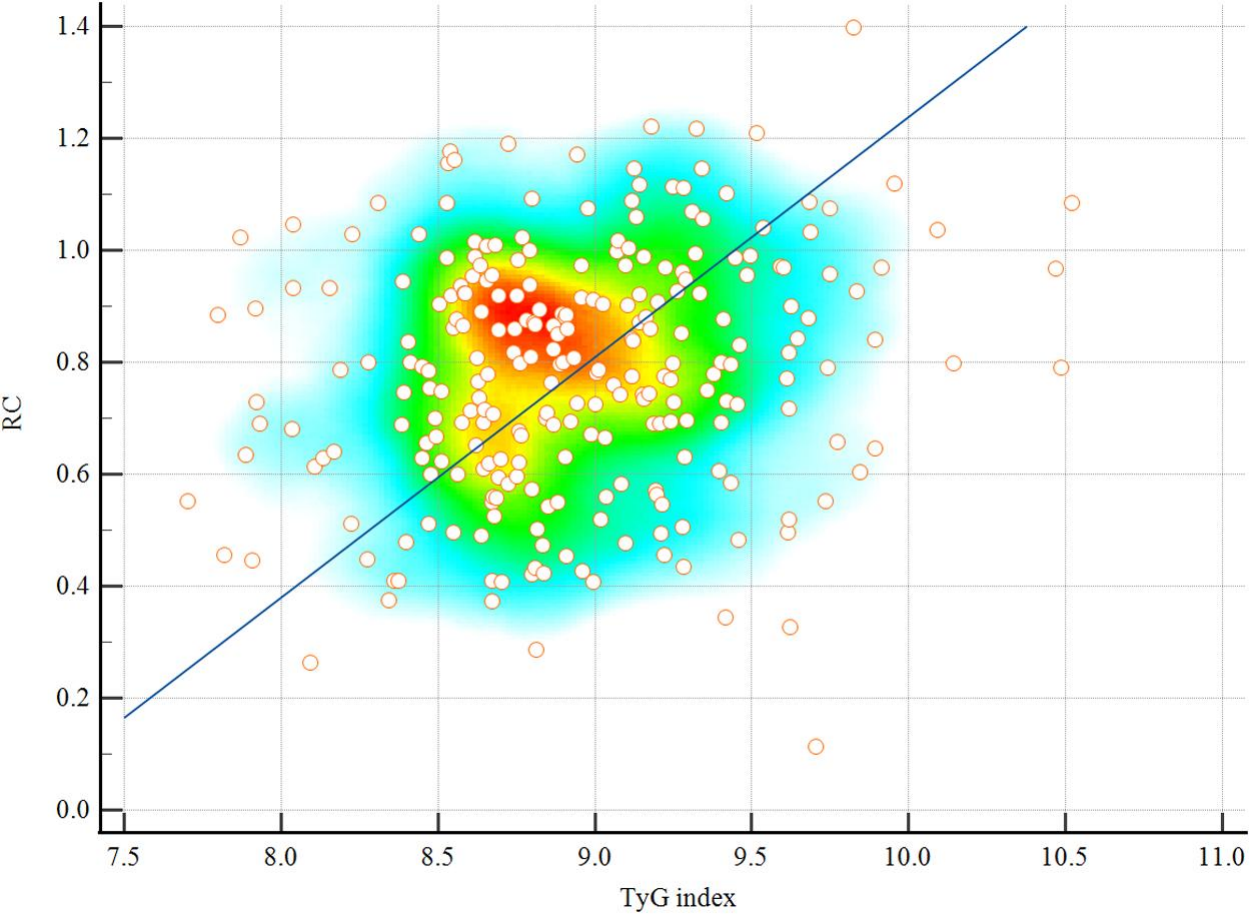
Scatter plot showing the Pearson correlation between the TyG index and RC in patients with CAD (*r* = 0.183, *P* = 0.006). A weak but statistically significant positive association was observed. Scatter plots are overlaid with density heatmaps, with linear regression lines shown. The color gradient from blue to red indicates increasing point density. TyG, triglyceride-glucose index; RC, remnant cholesterol; CAD, coronary artery disease.

To explore the relationship between the two biomarkers, Pearson correlation analysis was performed between the TyG index and RC within the CAD cohort. The results demonstrated a weak but statistically significant positive correlation (*r* = 0.183, *P* = 0.006), indicating that these markers are partially interrelated but likely reflect distinct underlying metabolic processes. This finding supports their use as complementary indicators in the cardiometabolic evaluation of young CAD patients. A scatter plot illustrating this correlation is presented in Figure 4.

### Correlation between metabolic parameters and TyG/RC levels

As shown in Table 5, the TyG index was strongly correlated with TG (*r* = 0.82, *P* < 0.001) and FPG (*r* = 0.67, *P* < 0.001), and showed a moderate negative correlation with HDL-C (*r* = –0.40, *P* < 0.001). RC was moderately to strongly correlated with TG (*r* = 0.64, *P* < 0.001), TC (*r* = 0.70, *P* < 0.001), LDL-C (*r* = 0.56, *P* < 0.001), and ApoB (*r* = 0.51, *P* < 0.001), and negatively correlated with HDL-C (*r* = –0.45, *P* < 0.001). These findings indicate that while TyG primarily reflects glucose–lipid dysregulation, RC is more closely linked to cholesterol-rich lipoproteins.

**Table 5:** Correlation coefficients (r) and *P* values between the TyG index, RC, and metabolic parameters.

Variables	TyG index (r)	TyG *P* value	RC (r)	RC *P* value
TG	0.82	<0.001	0.64	<0.001
FPG	0.67	<0.001	0.20	0.01
LDL	0.10	0.15	0.56	<0.001
HDL	−0.40	<0.001	−0.45	<0.001
TC	0.15	0.04	0.70	<0.001
ApoB	0.12	0.07	0.51	<0.001

TyG, triglyceride-glucose index; RC, remnant cholesterol; TG, triglycerides; FPG, fasting plasma glucose; ApoB, apolipoprotein B; LDL, low-density lipoprotein; HDL, high-density lipoprotein; TC, total cholesterol.

## Discussion

This study evaluated the utility of the TyG index and RC in the detection and stratification of CAD among individuals aged ≤45 years. The findings showed that both TyG and RC were independently associated with the presence of CAD and the extent of multi-vessel involvement. Notably, RC showed better discriminative performance for identifying CAD, whereas TyG was more strongly associated with multi-vessel disease. These results underscore the complementary value of TyG and RC as accessible and non-invasive biomarkers for early risk evaluation of CAD in younger adults.

### Role and implications of the TyG index

In recent years, the prevalence of CAD among young adults has demonstrated a marked upward trajectory. This trend has been largely attributed to the increasing incidence of conventional cardiovascular risk factors in this age group, including hypertension, diabetes mellitus, tobacco use, and obesity (17, 18). Among these, IR has emerged as a critical pathophysiological driver in the initiation and progression of atherosclerosis. Tian et al. reported that, in comparison to older populations, IR and diabetes rank among the most powerful predictors of early-onset cardiovascular events in younger individuals (19). Consequently, targeting IR may offer a promising avenue for preventing premature CAD.

The TyG index—calculated using fasting plasma glucose and triglyceride levels—has gained widespread recognition as a feasible and accurate proxy for IR. Accumulating evidence supports its clinical relevance. For instance, one study demonstrated that elevated TyG index levels were predictive of incident atrial fibrillation following acute myocardial infarction (AMI) (20), while another study reported a positive correlation between the TyG index and the extent of coronary atherosclerosis in asymptomatic patients with type 2 diabetes (21). Consistent with these findings, our study revealed that the TyG index was significantly associated with CAD in young adults and served as an independent predictor of MVD.

The underlying mechanism likely involves IR-mediated endothelial dysfunction, smooth muscle proliferation, and chronic inflammation. In a prospective study of 6,028 patients, Wu et al. found that each unit increase in TyG was associated with a 22% higher risk of atherosclerosis progression—outperforming fasting glucose or triglycerides alone in predictive power (22). Similarly, in patients with type 2 diabetes undergoing PCI, TyG emerged as an independent prognostic marker for adverse cardiovascular outcomes (23).

The relevance of the TyG index in younger cohorts has been substantiated by multiple longitudinal investigations. Xu et al. conducted a follow-up study involving 4,573 individuals under 30 years of age and found that elevated TyG levels were strongly associated with future cardiovascular events and increased all-cause mortality (24). Another prospective study with a 7.4-year follow-up in healthy adults under 40 demonstrated that participants in the highest TyG quartile had a 1.258-fold increased risk of myocardial infarction compared to those in the lowest quartile, with TyG outperforming fasting glucose and TG in predictive value (25). With respect to disease burden, several studies have confirmed a link between elevated TyG and more extensive coronary artery involvement. For instance, in a cohort of 2,792 patients with CAD, individuals in the top TyG tertile had a 1.5-fold higher risk of MVD relative to those in the lowest tertile (26). Another comparative analysis showed that each incremental rise in TyG was associated with a 1.897-fold increase in coronary artery calcification scores and a 1.213-fold greater likelihood of MVD in patients presenting with acute coronary syndrome (7).

In alignment with these observations, our results indicated that TyG index values were significantly higher in patients with MVD compared to those with single-vessel disease. Logistic regression analyses confirmed TyG as an independent predictor of both CAD presence and multi-vessel involvement. Additionally, ROC curve analysis revealed that TyG outperformed RC in identifying MVD, suggesting its particular strength in assessing disease severity.

In our cohort, TyG index values were significantly higher in MVD patients and remained an independent predictor after adjustment. ROC analysis further showed that TyG outperformed RC in identifying multi-vessel disease, supporting its value in stratifying disease severity.

### Significance and mechanistic role of remnant cholesterol

RC, a lipid parameter that has gained prominence in recent years, is increasingly recognized for its strong association with atherosclerotic cardiovascular disease (ASCVD) development (27). RC is typically estimated by subtracting HDL-C and LDL-C from TC, thereby reflecting the cholesterol content of TRLs, including IDL, VLDL, and chylomicron remnants in the postprandial state. Unlike traditional lipid markers, RC captures atherogenic cholesterol components not accounted for by standard panels and is believed to explain a portion of the residual cardiovascular risk in individuals with well-managed LDL-C levels (28).

The European Atherosclerosis Society (EAS) has acknowledged RC as a clinically significant predictor of vascular events, with its pathogenic role supported by mechanistic studies (29). Following endothelial injury, RC-enriched particles infiltrate the intimal layer of the vasculature and are absorbed by macrophages and smooth muscle cells. These particles contribute to oxidative stress, endothelial dysfunction, local inflammation, foam cell generation, and vascular remodeling—processes integral to atherogenesis. Crucially, RC is now considered a key determinant of residual ASCVD risk, with several studies demonstrating that elevated RC levels are associated with increased cardiovascular events even when LDL-C levels are within target range (10, 28). Mechanistically, RC-containing lipoproteins can penetrate the arterial wall, undergo TG hydrolysis, and promote foam cell formation—processes that accelerate plaque progression and destabilization (29). Epidemiological evidence further reinforces the link between RC and atherosclerotic events. In a large population-based study, Varbo et al. demonstrated that each 1 mmol/L (39 mg/dl) increase in non-fasting RC was independently associated with a 2.8-fold elevated risk of ischemic heart disease (30). Similarly, Wadström et al. found that high RC levels significantly increased the likelihood of myocardial infarction, ischemic stroke, and peripheral artery disease, supporting its systemic pro-atherogenic effects (31).

Our findings are consistent with prior reports, showing that RC levels were significantly higher in young adults with CAD and independently associated with both disease presence and severity. ROC analysis revealed an AUC of 0.773 for RC in predicting CAD, surpassing the diagnostic performance of the TyG index and demonstrating superior sensitivity and specificity. However, when predicting multi-vessel disease, RC yielded an AUC of 0.683, which was lower than that of TyG. This suggests that while RC may be more adept at detecting disease presence, TyG could serve as a better marker of disease severity. Recent large-scale investigations provide further validation for the independent prognostic utility of RC. A 2021 European cohort study demonstrated that elevated RC levels increased ASCVD risk irrespective of LDL-C or ApoB concentrations. Interestingly, patients with elevated LDL-C but low RC had attenuated cardiovascular risk, whereas those with high RC and low LDL-C exhibited increased risk (14). Furthermore, a Mendelian randomization analysis involving nearly one million individuals confirmed a genetic causal relationship between elevated RC and increased risks of CAD, myocardial infarction, and stroke (OR = 1.51, 95% CI: 1.42–1.60, *P* < 0.001) (32).

Collectively, these findings indicate that RC functions not only as a marker of residual lipid-related risk but also as a biologically active and genetically validated driver of atherosclerosis. Our study adds novel evidence by establishing the independent predictive value of RC specifically in a young adult population. These results underscore the importance of incorporating RC assessment into clinical practice, particularly for individuals with borderline or controlled LDL-C levels who remain at elevated metabolic and cardiovascular risk.

### Comparison with previous studies and clinical implications

Our findings both reinforce and expand upon prior research regarding metabolic biomarkers and their relevance to CAD. While earlier studies have established associations between the TyG index, RC, and elevated cardiovascular risk, such investigations have predominantly focused on middle-aged or elderly populations, frequently within the context of established metabolic disorders such as diabetes, obesity, or clinically evident cardiovascular disease (19, 26, 27). By contrast, the present study specifically targets adults aged ≤45 years, thereby addressing a critical gap in the literature related to early-onset atherosclerosis. This younger demographic is increasingly affected by metabolic dysfunction but remains underrepresented in traditional cardiovascular risk stratification models.

Moreover, few existing studies have simultaneously examined TyG and RC within the same cohort. Our comparative analysis revealed that these markers offer complementary information: RC exhibited greater sensitivity in detecting the presence of CAD, whereas TyG demonstrated a stronger association with disease severity, particularly in cases involving MVD. This dual-marker strategy offers enhanced resolution for risk assessment—particularly relevant for younger individuals who may not present with elevated traditional lipid measures such as LDL-C. Although current clinical guidelines prioritize LDL-C as the primary target for lipid-lowering therapy, recent evidence suggests that significant residual cardiovascular risk may persist even with optimal LDL-C control. RC may help explain this residual risk and therefore deserves consideration as an additional therapeutic and diagnostic target (14, 28).

From a clinical standpoint, our findings support the integration of both TyG and RC into existing cardiovascular risk assessment frameworks, especially for young patients with borderline lipid profiles or unexplained symptoms suggestive of CAD. Both markers can be derived from routinely available fasting glucose and lipid panels, making them practical for widespread application in primary care and specialized settings. For instance, the TyG index may help identify patients at increased risk for MVD, thereby guiding decisions for earlier diagnostic imaging or the initiation of more intensive therapeutic interventions. Concurrently, RC can complement LDL-C measurements in detecting patients with residual atherogenic risk who may benefit from additional lipid-modifying therapies or targeted lifestyle interventions. Furthermore, our findings have potential implications for future cardiovascular risk prediction models. Widely used algorithms, such as the Framingham Risk Score or the Pooled Cohort Equations, may underestimate cardiovascular risk in younger adults, particularly those without overt dyslipidemia. Incorporating TyG and RC into existing models—or developing new, age-specific tools—may enhance risk detection, support earlier intervention, and ultimately improve long-term outcomes.

Notably, this study also identified ROC-derived cutoff values for both TyG and RC that may have practical utility in clinical settings. For example, a TyG index threshold of 8.893 and an RC threshold of 0.728 mmol/L were found to offer reasonable sensitivity and specificity for identifying CAD in young adults. These thresholds, derived from a relatively healthy and underdiagnosed population, may help flag individuals who do not yet meet traditional lipid or glucose criteria for intervention but still carry significant cardiometabolic risk. While these cutoffs align with ranges reported in studies of older or diabetic populations (21, 24, 28), their application in younger adults warrants further validation. Future prospective studies should explore whether incorporating these specific thresholds into screening protocols could improve early detection and preventive strategies in clinical practice.

Interestingly, our correlation analysis revealed a weak but statistically significant positive association between the TyG index and RC (*r* = 0.183, *P* = 0.006) among CAD patients. This suggests that although both markers are linked to metabolic dysregulation, they may reflect distinct yet overlapping pathways—TyG being more indicative of insulin resistance and glucose-lipid imbalance, while RC represents remnant lipoprotein-driven atherogenesis. Their modest correlation supports the notion that they offer complementary, rather than redundant, information. This further validates the utility of incorporating both markers in risk stratification models for young adults with suspected CAD.

In summary, this study highlights the potential clinical value of the TyG index and RC as cost-effective, non-invasive biomarkers for identifying cardiovascular risk in young adults. Their distinct yet complementary associations with the presence and severity of CAD suggest that their combined use may enhance risk stratification and support more tailored preventive approaches in this increasingly vulnerable population.

### Limitations

Despite the valuable contributions of this study, several limitations should be considered. First, the retrospective and single-center nature of the study introduces potential selection bias and restricts the generalizability of the findings. In particular, local referral practices and institutional criteria for performing CAG in young adults may differ from those in other centers, potentially influencing patient selection and limiting the applicability of our results to broader populations. Future validation through larger, multicenter, prospective studies is necessary to strengthen external applicability. Second, while the TyG index and RC were calculated using routinely collected laboratory data, insulin resistance was not directly assessed using gold-standard methods such as the hyperinsulinemic-euglycemic clamp technique. As a result, the TyG index may not fully encapsulate the complexity and variability of insulin resistance in all individuals. Third, although multivariate adjustments were performed, residual confounding cannot be completely excluded. Important lifestyle variables such as dietary intake, physical activity, and family history of premature CAD were not captured in this retrospective dataset. These factors could influence both TyG/RC levels and CAD risk, and their omission may introduce unmeasured confounding. Fourth, the cross-sectional design limits the ability to establish temporal or causal relationships between TyG or RC levels and the development of coronary lesions. Fifth, while all participants were aged ≤45 years, we did not perform further stratification by age subgroups (e.g., ≤35, 36–40, 41–45) due to limited sample sizes and potential statistical underpowering. This may have limited the ability to detect subtle age-related metabolic variations within the young adult population. Sixth, RC was calculated using an indirect estimation formula rather than measured directly by ultracentrifugation or other reference laboratory methods. While this approach is commonly used in clinical research, it may introduce potential measurement error or bias. Lastly, because all participants in this study were Chinese, genetic background, ethnicity, and environmental influences were not examined, all of which may affect biomarker expression and their predictive significance across different populations.

## Conclusion

In summary, this study provides evidence that both the TyG index and RC are independently and complementarily associated with the presence and severity of CAD in adults aged ≤45 years. RC showed relatively higher sensitivity for identifying CAD, while the TyG index was more strongly associated with the extent of disease, particularly in cases of multi-vessel involvement. These findings highlight the potential value of incorporating TyG and RC into cardiovascular risk assessment frameworks, especially for younger patients presenting with non-specific symptoms or normal LDL-C levels. As readily accessible, low-cost, and non-invasive biomarkers, TyG and RC may offer practical utility in supporting early risk identification and informing individualized management strategies for premature atherosclerosis. Further prospective studies and interventional trials are needed to confirm these findings and to evaluate their applicability in clinical decision-making and preventive practice.

## Data Availability

The raw data supporting the conclusions of this article will be made available by the authors, without undue reservation.
